# Non-violent resistance parental training versus treatment as usual for children and adolescents with severe tyrannical behavior: a randomized controlled trial

**DOI:** 10.3389/fpsyt.2023.1124028

**Published:** 2023-05-04

**Authors:** Erica Fongaro, Safa Aouinti, Marie-Christine Picot, Florence Pupier, Haim Omer, Nathalie Franc, Diane Purper-Ouakil

**Affiliations:** ^1^Centre Hospitalier Universitaire de Montpellier, Saint Eloi Hospital, Montpellier, France; ^2^CESP INSERM U 1018 UVSQ Psychiatry Development and Trajectories, Villejuif, France; ^3^Centre Hospitalier Universitaire de Montpellier, Unité de Recherche Clinique and Epidémiologie, DIM, Montpellier, France; ^4^Department of Psychology, Tel Aviv University, Tel Aviv, Israel

**Keywords:** parent training, parent–child interaction, behavior problems, coping, oppositional defiant disorder

## Abstract

**Objective:**

This single-blinded, randomized, parallel group superiority trial evaluates whether the Non-Violent Resistance (NVR) program, a 10-session parental-group intervention, was more effective in reducing stress in parents of children aged 6–20 years and displaying severe tyrannical behavior (STB) compared to a treatment as usual (TAU) intervention that provided supportive counseling and psychoeducation.

**Methods:**

Eighty two parents of youth aged 6–20 years with STB were enrolled by the Child and Adolescent Psychiatry Department at the University Hospital of Montpellier (France). A random block and stratified by age (6–12 and 13–20 years) randomization, was performed. All participants were interviewed by independent, blinded to group assignments, research assistants, and completed their assessments at baseline and treatment completion (4 months from baseline). Since this program has not been previously evaluated in this population, the study primarily evaluated the efficacy, using the Parenting Stress Index/Short Form (PSI-SF). The primary outcome was the change from baseline to treatment completion of the PSI-SF total score.

**Results:**

Seventy three participants completed the study and were available for analysis (36 NVR and 37 TAU). At completion, between-groups comparison of the change (completion minus baseline) in the total score of PSI-SF was not significant (NVR: −4.3 (± 13.9); TAU: −7.6 (± 19.6); two-sample *t*-test *p* = 0.43; effect size of −0.19 [−0.67, 0.28]).

**Conclusion:**

Contrary to our expectation, NVR was not superior to TAU in reducing parental stress at completion for parents of children with STB. However, NVR showed positive outcomes in the follow-up, pointing to the importance to implement parental strategies and following this population over longer time periods in future projects.

**Clinical trial registration**: Clinicaltrials.gov, identifier NCT05567276.

## Introduction

### Background

Family violence is usually perceived as including mainly child abuse or intimate partner violence. However, child-to-parent aggressive behavior is another understudied facet of domestic violence. Although it has received limited attention until recent years, it is far from being an uncommon phenomenon (1–4). Prevalence rates of child-to-parent violence (CPV), defined by aggression perpetrated at least once in the last year, range from 5 to 21% for physical violence, and 33 to 93% for psychological violence (3, 5). The variety of definitions and measurements used to describe CPV, may explain high discrepancies in reported prevalence (4). Data suggest that adolescents displaying aggressive behavior toward their parents also have violent behavior outside the family (6). Risk factors reported for CPV are similar to those of overall aggressive behavior in adolescents: low socioeconomic status, coercive education, child neglect and abuse and intimate partner violence (2, 7–10).

The present study focuses on a specific type of CPV described as severe tyrannical behavior (STB) (11). STB in children is described as “Oppositional Defiant Disorder with family tyranny” by several clinicians (12, 13) because oppositional defiant disorder (ODD) symptoms in those children are confined to only one setting: the home. Intra-familial ODD (IODD) is considered a mild form of ODD by the DSM-5. STB is a family scenario characterized by an inversion of the family hierarchy, with the child dominating and controlling the family dynamics through child-to-parent psychological and sometimes physical violence. Parents gradually lose control over the situation and accommodate the difficult behavior, by sacrificing their well-being and basic needs, in fear of their own child’s reactions. Any attempt to resist the child’s behavior may trigger violence, tantrums, suicidal threats, or self-harm, leaving the parents helpless and desperate. Children and adolescents with STB show strong resistance to change and poor help-seeking behaviors. The STB family scenario is often disclosed late in the help-seeking process because parents feel ashamed and fear social judgment. Moreover, children with STB preferentially abuse their parents and close family members. Their normal social behavior outside home differentiates them from children with generalized forms of disruptive behavioral disorders such as oppositional defiant and conduct disorders (11). This accentuates the fact that parents rarely feel believed, and suffer isolation and guilt. In addition, while STB is closer to a family pattern than to a formal diagnosis, it is frequently associated with a variety of psychiatric conditions such as attention deficit hyperactivity disorder (ADHD), mood and anxiety disorders, and autism-spectrum disorders (7, 14, 15). Additionally, in the STB scenario, these conditions may be overlooked because of parental accommodation and fear of social stigma in both children and parents. STB shares features with adolescents at an “invisible” risk for psychopathology and suicidal behavior identified by Carli et al. (16). STB has also been associated with failure to successfully go through the developmental stage of emerging adulthood as it fosters dysfunctional dependence and entitlement and has a long-term impact on functioning (17).

Mental health professionals dealing with STB scenarios face various challenges: they have to encourage parents’ communication about family violence, screen for underlying psychiatric conditions in children and build parental skills to change the family dynamics.

Since children with STB usually lack motivation to engage in therapy, we have set up a parent training program based on Non-Violent Resistance (NVR) strategies, with the aim of influencing parental behavior as a key feature in the therapeutic process. NVR has been developed by Haim Omer to help families facing problematic behaviors (18–23) and entrenched dependence in their children (24). NVR parent training focuses on non-escalating coping responses to the child’s violence, de-accommodation, and self-control. Moreover, the program helps parents to step out from secrecy and social isolation by building a support network.

NVR represents an innovative approach in psychotherapy that fosters societal adaptations to parent–child relationships and promotes a new model of parental authority. Specifically, it has been proven effective in supporting parents of children with ADHD (25) and anxiety disorders (26), self-destructive or aggressive behaviors of youth (27, 28) and highly dependent young adults (29). Despite these promising findings, there are no data on NVR therapy involving parents of children with STB, a distressed parent population affected by social stigma from a perceived loss of parental authority and in need of efficient coping strategies (13).

### Objectives

The current study evaluates whether the NVR program, a protocolized 10-session parental-group intervention, was more effective in reducing stress in parents of children aged 6–20 years and displaying STB compared to a treatment as usual (TAU) intervention that provided supportive counseling and psychoeducation. Since this program has not been previously evaluated in this population, the main outcome of this study is efficacy of the NVR program, using the Parenting Stress Index (PSI) (30). Additionally, the study aims to investigate the efficacy of NVR on parental anxiety and depression and children’s behavioral symptoms as secondary outcomes.

## Materials and methods

### Study design

This was a single-blinded, randomized, parallel group superiority trial that compared the NVR intervention group with a TAU group during a 4-month period. After this timeframe, parents of the TAU group were also offered to participate in the NVR program. The study was conducted within the context of a clinical treatment aimed at providing a non-pharmacological intervention for parents of children with STB. All screened youth and families were offered services regardless of the study participant agreement. The study was approved by the ethical committee (*Comité de Protection des Personnes Ouest III*).

The study recruitment occurred from July 2017 to March 2019 with follow-up interviews completed by July 2019.

### Participants

Eighty two parents of youth aged 6–20 years were enrolled by the Child and Adolescent Psychiatry Department in Saint-Eloi University Hospital in Montpellier (France). Inclusion criteria were: (a) being the parent of a child aged 6–20 years who endorses the DSM-5 criteria of IODD; (b) two or more positive answers to the *custom* questions routinely used to identify STB in children/adolescents at the University Hospital of Montpellier, related to a 12-month period:

▪ Are you afraid of your child?
▪ Is your child physically or psychologically violent toward you?
▪ Do you think that your child is the decision maker in the family?
▪ Do you feel ashamed by your situation at home?

(c) being able to attend at least half of the sessions.

Exclusion criteria were the following: (a) predicted absence from at least half of the sessions; (b) subject deprived of liberty, (c) subject under guardianship or curatorship, and (d) lack of written informed consent from parent.

### Procedure

Participants were recruited at both local and national levels using different methods, including electronic *media*, print advertising, internal volunteer lists and physician referrals.

Eligible parents were informed by their referent mental health professionals about this study. In other cases, an STB patient organization provided information about the study to their members and these parents directly applied for the study. Once the clinician identified eligible parents and children, they received detailed written and verbal information about the study. After signing informed consent, parents meeting the criteria were invited for the inclusion visit.

The inclusion visit was conducted by a trained research assistant in charge to complete baseline characteristics. The visit also aimed at assessing the child’s medical and psychological history, as well as STB and how it was shaping the family dynamics. The NVR program was explained to participants based on a manual published in 2017 and developed by the principal investigator of this study, Franc and Omer (11). After consent, participants were randomized to the NVR or TAU condition.

Parents in the NVR group received the 10 2-h sessions, over a period of 4 months. The NVR protocol was provided by clinicians trained in the program. TAU received non-pharmacological and pharmacological treatments as usually provided. All participants were interviewed by independent research assistants and completed their assessments at baseline and following their last treatment session (4 months from baseline). Only the participants of NVR were asked for a 4-month post-treatment evaluation (8 months from baseline), thus excluding a follow-up comparison with TAU.

Both parents were allowed to follow the program, but only one of them could complete the questionnaires.

### Outcomes

Independent evaluators, blinded to group assignment, administered the self-report measures and conducted the semi-structured interviews listed in Table 1 at pretreatment (inclusion visit, V0), completion (4 months from baseline, V1) and 4-month follow-up (8-month from baseline, V2). All clinician/parent/teacher-rated measures were completed at V0 and V1, unless otherwise noted. Only the NVR group, completed the V2 measures.

**Table 1:** Participant timeline.

Visit	Inclusion visit (V0)	Randomization	Visit 1 (V1)	Visit 2 (NVR only, V2)
Date	- 2 months	Day 0	+ 4 months	+ 8 months
Inclusion criteria	x			
Randomization		x		
Parental- reported ISP-SF, HAD	x		x	x
Parental/Teacher-reported CBCL, SDQ	x		x	x
K-SADS	x			
Social and medical data	x			

Time and events of the REACT study for the assessment of primary and secondary endpoints; V0 = inclusion visit; V1 = visit at treatment completion (4 months from baseline); V2 = visit post-treatment (8 months from baseline).

The primary endpoint variable was the change from baseline (inclusion visit) to completion of treatment (V1) in the total score of the Parenting Stress Index/Short Form (PSI-SF) (30). We hypothesized that the decrease in PSI-SF total score between baseline and end of treatment would be superior in NVR compared to TAU. Secondary outcomes concern the children’s behavior, parental anxiety and depression by examining pre-post changes and between-group changes at V1. We also examined changes in parental and child variables at 8 months from baseline and 4 months after completion of the NRV program (V2).

### Parenting stress index/short form

Parenting Stress Index/Short Form (PSI-SF) is a short version of the PSI by Abidin in 1995 (30) and is a 36-item, self-report measure of parenting stress. The French version was translated and validated by Bigras et al. (31). The PSI-SF has three subscales: Parental Distress, Parent–Child Dysfunctional Interaction, and Difficult Child. Each subscale consists of 12 items rated from 1 (strongly disagree) to 5 (strongly agree). A total score is calculated by summing the three subscales scores, ranging from 36 to 180. Scores of 90 or above indicate a clinical level of parental stress. Traditionally, the PSI-SF has been used to measure parenting stress in parents from clinical and high-risk populations (32–36), and to measure treatment effectiveness (37, 38). The PSI-SF was administered at baseline, at completion and for the NVR group also at follow-up.

### Hospital anxiety and depression scale

The Hospital Anxiety and Depression Scale (HADS) (39) is a 14-item measure designed to assess anxiety and depression symptoms in adults. Items are rated on a 4-point severity scale. The HADS produces a scale for anxiety (HADS–A) and for depression (HADS–D). To screen for parental anxiety and depressive symptoms, the following interpretation can be proposed for each of the scores: scores of 7 or less are considered no symptomatology; scores between 8 and 10 are considered doubtful symptomatology; scores of 11 and more are considered definite symptomatology. The French version was validated by Duquette (40). The HADS was administered at baseline, at completion and for the NVR group also at follow-up.

#### Child behavior checklist

The *Child Behavior Checklist* (CBCL) (41) is one of the most standardized parental reports to access youth internalizing and externalizing symptoms that describe their children’s behavioral and emotional problems within an environment according to age, gender and informant. The CBCL comprises a 113-item scale that utilizes T-scores to enable a comprehensive assessment of the children’s behavioral symptoms. Scores above the 98th percentile (T-scores >70) are considered to be in the clinical range, while for externalizing and internalizing problems are indicated by T-scores >64. Borderline elevations (over 95th percentile) range from 60 to 63 on the externalizing and internalizing problems and 65–69 on the other scales. It is translated into 110 languages, including French (42). The CBCL was administered at baseline, at completion and for the NVR group also at follow-up.

### Strengths and difficulties questionnaire

The parent/teacher-reported Strengths and Difficulties Questionnaire (SDQ) is a brief behavioral screening questionnaire used in clinical and research settings. The SDQ measures emotional and behavioral symptoms, and their impact on areas of functioning, called the impact score (43). The SDQ symptom part consists of 5 subscales, each containing 5 items. The scales measure emotional symptoms, conduct problems, hyperactivity-inattention, peer relationship problems, and prosocial behaviors. Cut-off scores are: total difficulties: “borderline” = 14–16; “abnormal” ≥ 17; emotional symptoms: “borderline” = 4; “abnormal” ≥ 5; conduct problems: “borderline” = 3; “abnormal” ≥ 4; hyperactivity-inattention: “borderline” = 6; “abnormal” ≥ 7; peer relationship problems: “borderline” = 3; “abnormal” ≥ 4; prosocial behaviors: “borderline” = 5; “abnormal” ≤ 4; impact scores (impact, family, learning, leisure activities, friendship and child’s distress): “borderline” = 1; “abnormal” ≥ 2.

SDQ has become an internationally recognized tool which is extensively used in both French research and clinical settings (44, 45). The parent-rated and teacher-rated SDQ were administered at baseline, at completion and for the NVR group also at follow-up.

### Intervention

#### Treatment

The NVR program included 10, 2-h sessions. Sessions took place over 4 months. The program was given by a psychiatrist and a psychologist. We implemented 5 NVR groups of up to 20 participants.

NVR is a parent training program that focuses on the well-being of parents in order to help them deal with their helplessness, isolation, and escalatory interactions with their children (27). The strategies for NVR group management are described in the manual book for clinicians: Franc and Omer “Accompagner les parents d’enfants tyranniques, un programme en 13 séances” (11).

Each session contained a theoretical and a discussion part. First, parents were invited to give feedback about what happened at home with their child during the last week, and what strategies they could use. After the first discussion between parents, a principle of NVR is described and therapists presented a NVR strategy to use at home (e.g., write a declaration, set up a sit-in). At the end, we encouraged a last discussion between parents about the main content of the session in order to give inputs for implementing and reinforcing effective strategies. The contents of each session are summarized in Table 2.

**Table 2:** Themes and contents of NVR program in the REACT study.

First session	Presenting the program: definition of STB and impact on family dynamic; creating a group dynamic through active interactions between parents and experience sharing;
Second session	Providing psychoeducation on psychopathology that could be involved in STB such as: anxiety disorders, ADHD, bipolar disorders, ASD, to help parents understand their child’s functioning and initiate appropriate clinical assessments (especially when referred by media)
Third session	Describing the model of “new authority” for parents which emphasizes self-control and persistence over control of the child, the pathways through which violence escalates, and how to have educative reactions.
Fourth session	Preparing and writing a declaration of non-violence that could be shared with the group by voluntary parents
Fifth session	Describing different strategies to regain control, such as setting a sit-in the child’s room
Sixth session	Understanding the course of temper tantrums and coping with emotional crisis without escalating; avoiding harm for the child and for self.
Seventh session	Developing and contacting a support network of minimum 10 persons, to inform them about the child’s STB so that the child may face social judgement and parents can get help
Eighth session	Self-care messages for parents; avoiding sacrifice statements; initiation to mindfulness.
Ninth session	Controlling the child’s use of screens that may be a particular source of conflict, with high risks of school-dropouts.
Tenth session	Concluding the program with positive messages: refusing to give in to special treatment for the child while promoting reconciliation acts and regaining authority.

#### Control group

In the control group, there was no specific intervention for parents. For 4 months, TAU continued to receive non-pharmacological (e.g., psychotherapies, psychosocial, or psychiatric interventions) and pharmacological therapies (e.g., anxiolytics, antidepressants) as usually provided. The TAU group participated in the assessments as described in the procedures. At the end of 4 months, these families could benefit from the NVR program.

### Statistical power and analyses

#### Sample size

In a randomized controlled trial evaluating an emotion coaching program (46) in parents of children with behavioral problems, the change at 3 months in the parental stress index was −13 in the intervention group compared to +2 in the control group. We assumed a variation at 4 months of −13 points in the NVR group versus 0 in the control group. The number of subjects required was estimated at 29 subjects per group, with an SD of 15 (46), a first-order risk of 5% and a power of 90% under a two-sided hypothesis. Considering the possible discontinuation of follow-up, the number of subjects included increased by 10%. We included 82 subjects (40 in TAU and 42 in NVR).

#### Randomization

Randomization was done using Ennov Clinical^®^ online software, few days before the start of the group sessions. In order to construct balanced NVR and TAU groups on age, a randomization, stratifying by age (6–12 years and 13–20 years) with blocks of variable sizes was performed. The study was not double-blinded since the families knew whether they received specific care or not. No independent evaluator was required for any of the outcome measures. The questionnaires answers were integrated by the investigator into the online case report form.

Adherence to the NVR program was calculated as the percentage of parents that attended at least at 8 out of 15 sessions.

### Statistical analysis

The analysis of the primary and the secondary outcomes were performed on the Full Analysis Set (FAS) that will include all subjects who are randomized and have a valid primary efficacy measurement.

The baseline characteristics of children and parents was described with proportions for categorical variables and with means and standard deviations (SD) for quantitative variables. The comparability of the 2 groups was checked for all these baseline characteristics.

The change from pre- to post-treatment on the main and secondary outcomes were compared between groups using a two-sample *t*-test. Changes in PSI-SF, CBCL and SDQ scores for the NVR group were also analyzed using mixed linear models with a subject-specific random intercept. The fixed effect was the visit or time effect (at baseline, 4- and 8-months). The absolute mean or median difference and its 95% confidence interval were calculated using Hodges-Lehmann Method. The estimated Cohen’s d effect size and its precision 95% confidence interval (CI) were calculated for the main and secondary outcomes. The effect sizes magnitude were classified as: ≥ −0.15 and < 0.15 “negligible,” ≥ 0.15 and < 0.40 “small,” ≥ 0.40 and < 0.75 “medium,” ≥ 0.75 and < 1.10 “large,” ≥ 1.10 and < 1.45 = “very large,” and ≥ 1.45 “huge” (47).

In order to study the persistence of the effect on parental stress, anxiety and depression, the changes of the PSI-SF and HADS between baseline and completion in both groups, and baseline-follow up and completion—follow up in the experimental group was analyzed using paired *t*-test or Paired Wilcoxon test.

Statistical analyzes were implemented using SAS (Enterprise Guide, version 8.2; SAS Institute; Cary, North Carolina, United States) (48) by the Clinical Research and Epidemiology Unit of the Montpellier University Hospital. A two-sided value of *p* < 0.05 was considered to indicate statistical significance.

## Results

### Participants

**Figure 1:**
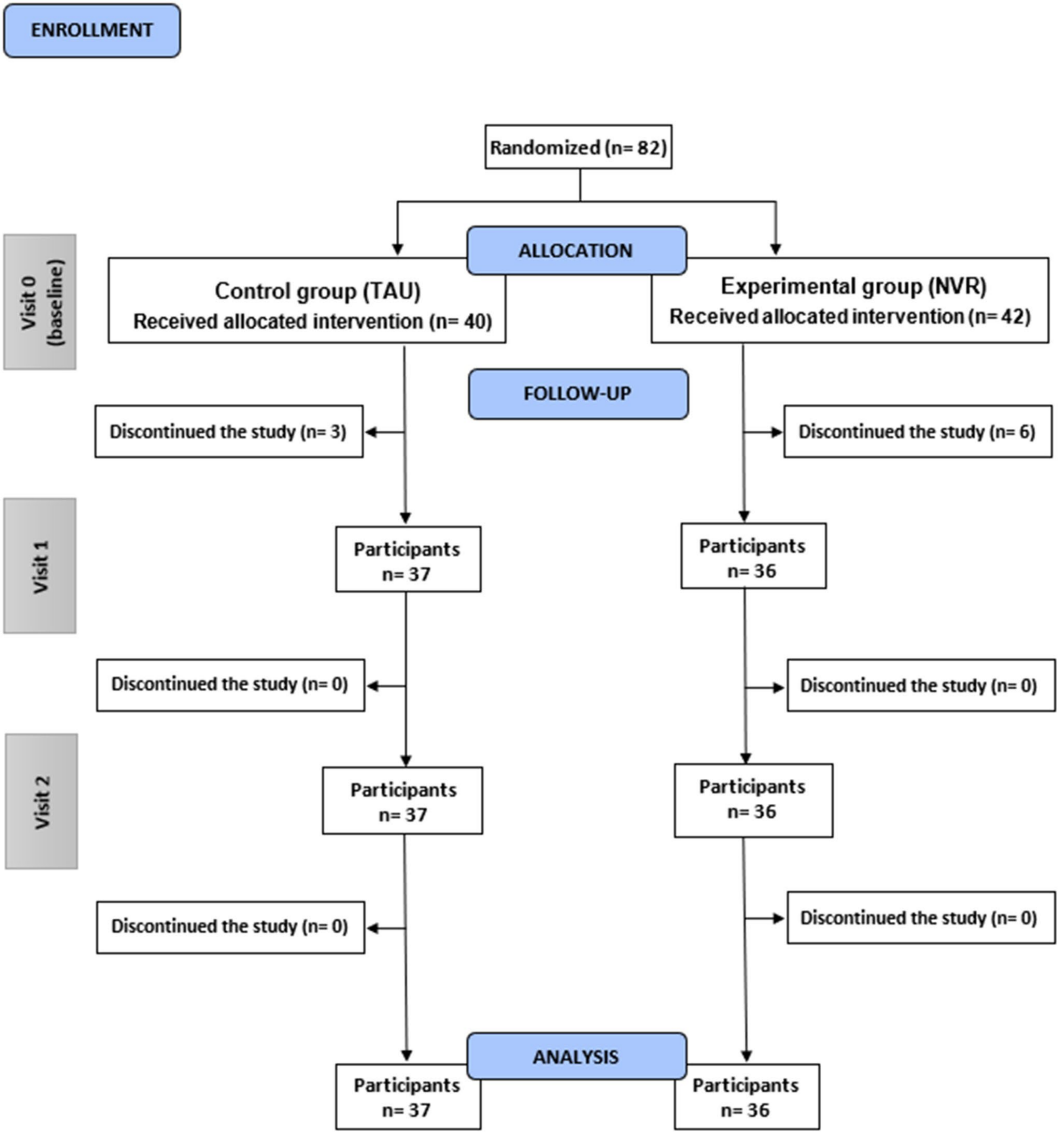
Consolidated Standards of Reporting Trials (CONSORT) flow diagram of the REACT study. Visit 0 (inclusion visit); Visit 1 (completion); Visit 2 (follow-up). Number of participants (n). Control group (TAU); Experimental group (NVR).

**Figure 2:**
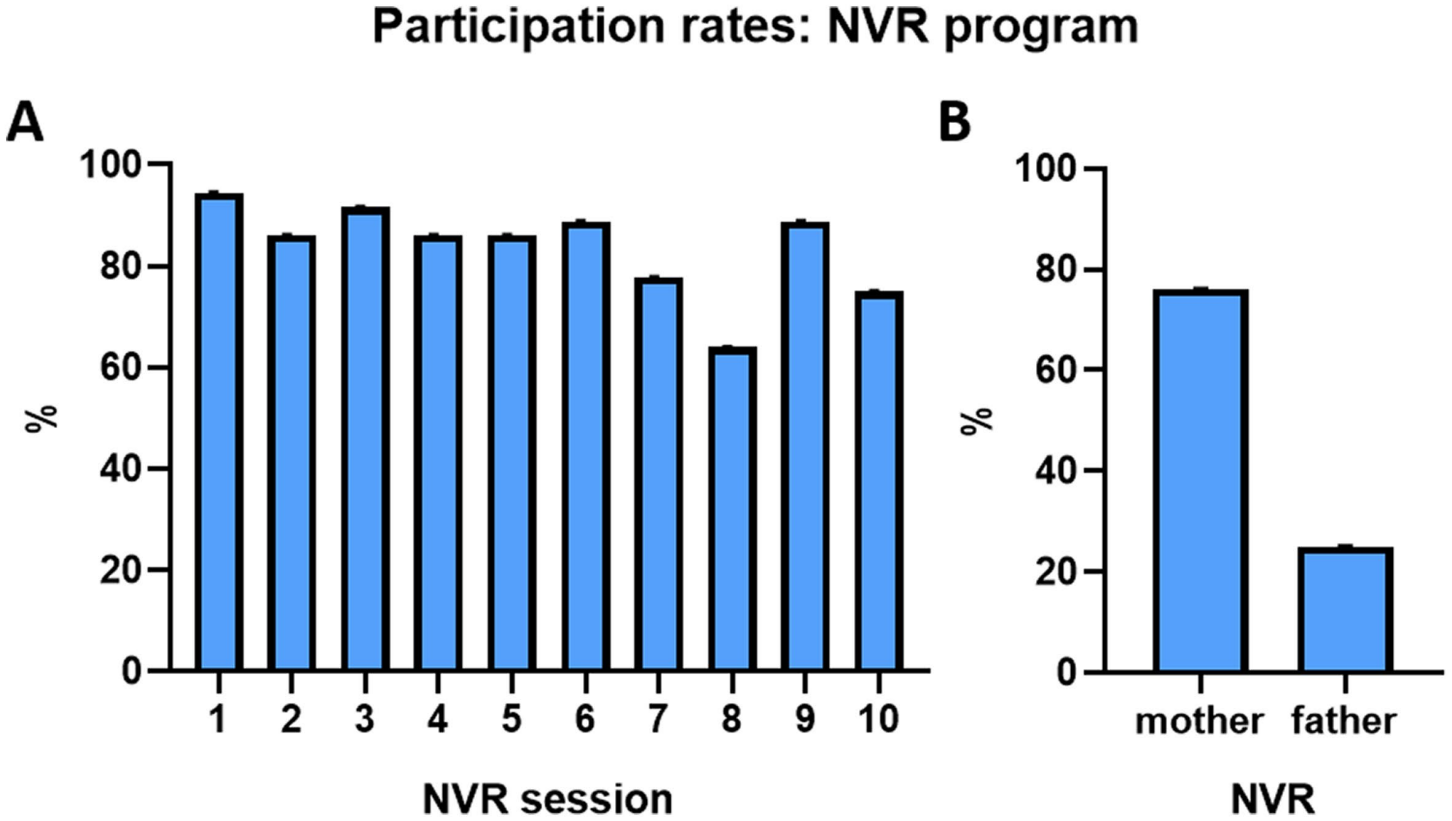
Participation rates in the NVR program. **(A)** Participation rate for each NVR session. **(B)** Attendance of the NVR program by mothers and fathers.

**Figure 3:**
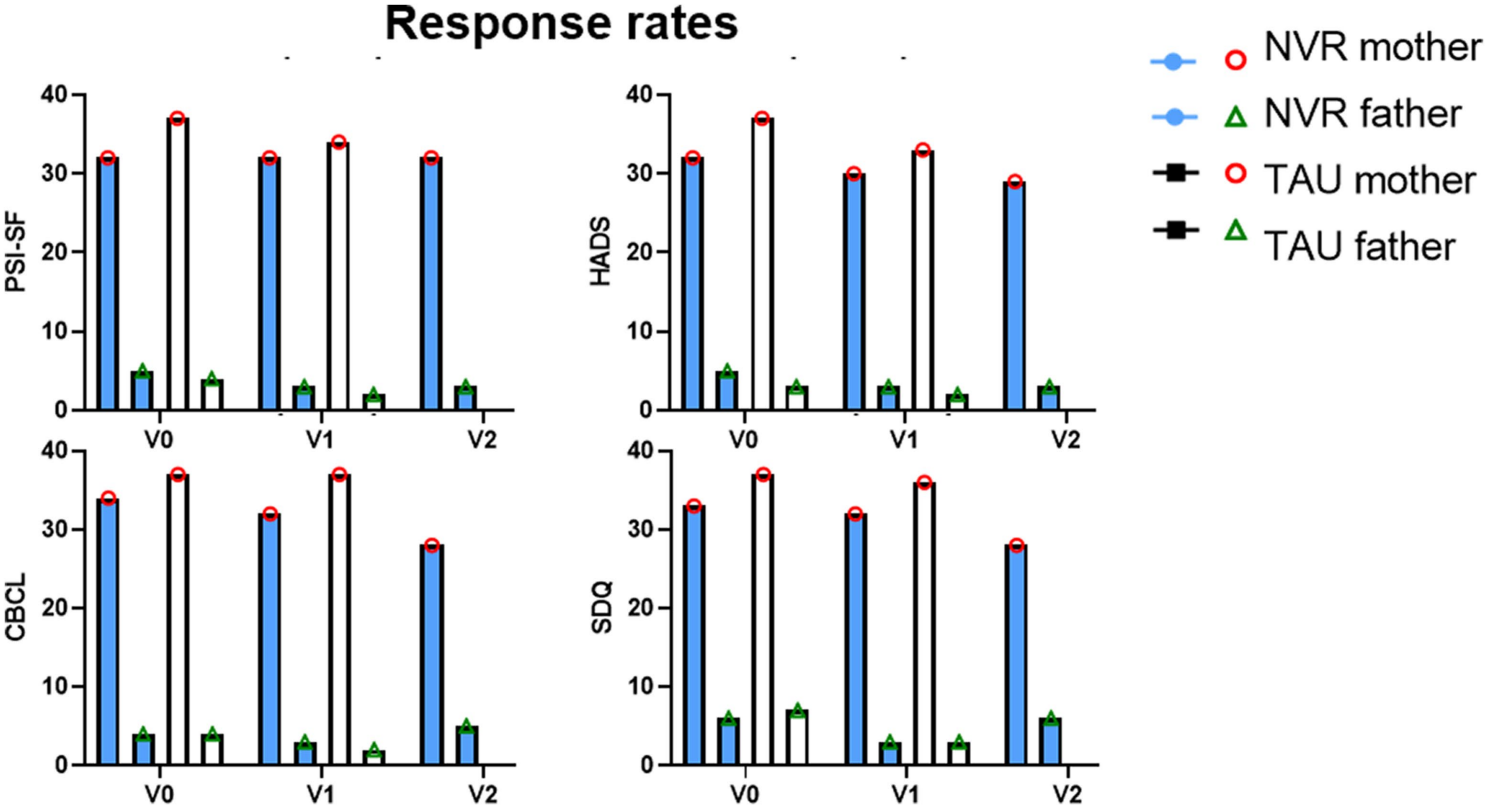
Response rates. Response rates by mothers and fathers. Visit 0 (inclusion visit); Visit 1 (completion); Visit 2 (follow-up).

Among the 82 families included nine participants were excluded following randomization and baseline assessment and did not return for treatment sessions or additional research interviews (participants’ flow chart is presented in Figure 1). In both groups the delay of randomization and the initiation of intervention was of 2 months. Six families from NVR (14.3%) and 3 from TAU (7.5%) discontinued the study (*p* = 0.05) for mainly medical and logistical reasons (NVR: one did not come back after multiple calls, two of them had an improvement/severe deterioration of symptoms, three had organizational or travel-related mobility problems; TAU: two did not reach out after multiple calls, one had organizational issues). A total of 73 participants completed the study and were analyzed (36 NVR and 37 TAU) with a mean (± SD) participation rate in the NVR sessions of 76% (± 28.6) for mothers and 25% (± 35.5) for fathers, considering both parents could be present at the session (Figure 2B). The adhesion rate stayed high throughout the whole intervention period, despite the length of the program (see Figure 2A). The majority of family members participating and completing the assessments were mothers in both groups (Figures 2B, 3). Since father attendance was not sufficient to allow a comparative analysis between groups, we used and compared only mothers’ results (when both questionnaires were available, we used mothers’ scores).

### Baseline demographic and clinical characteristics

**Figure 4:**
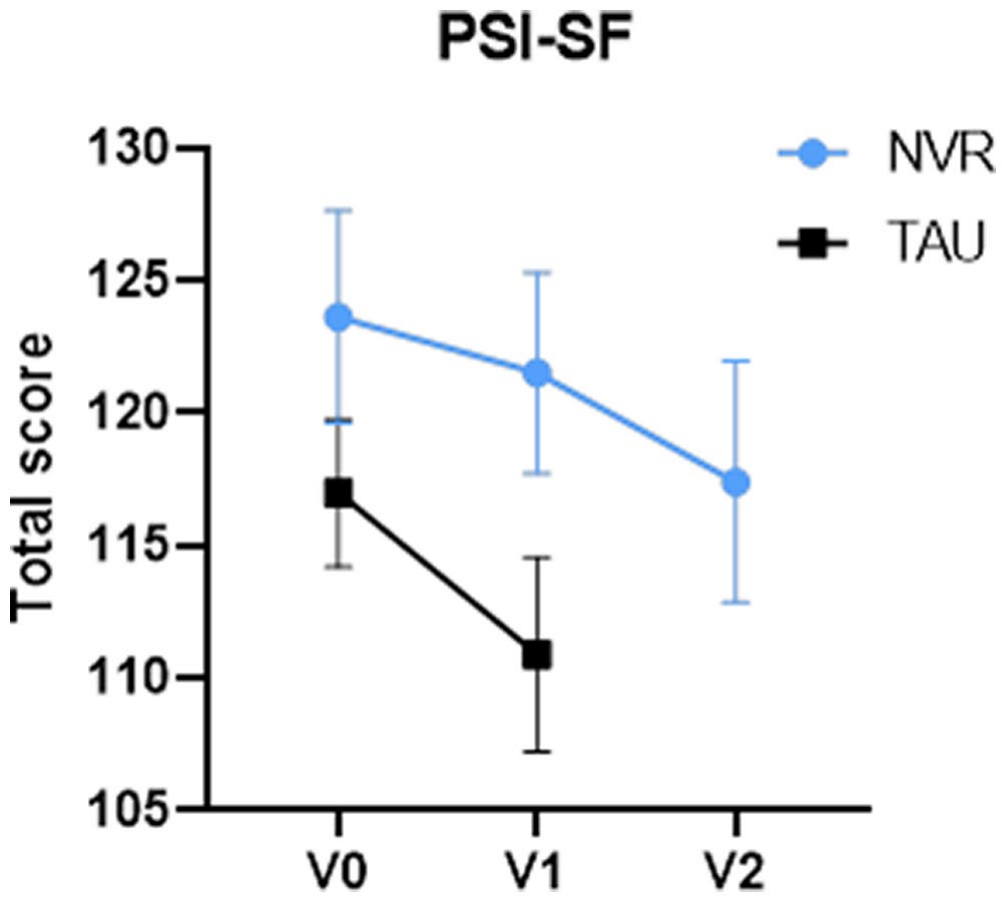
PSI-SF total scores. Values of PSI-SF for number of subjects at baseline, V0 (NVR = 32, TAU = 37), at completion, V1 (NVR = 32, TAU = 34) and at post-treatment, V2 (NVR = 32). Values are mean ± standard deviation.

**Figure 5:**
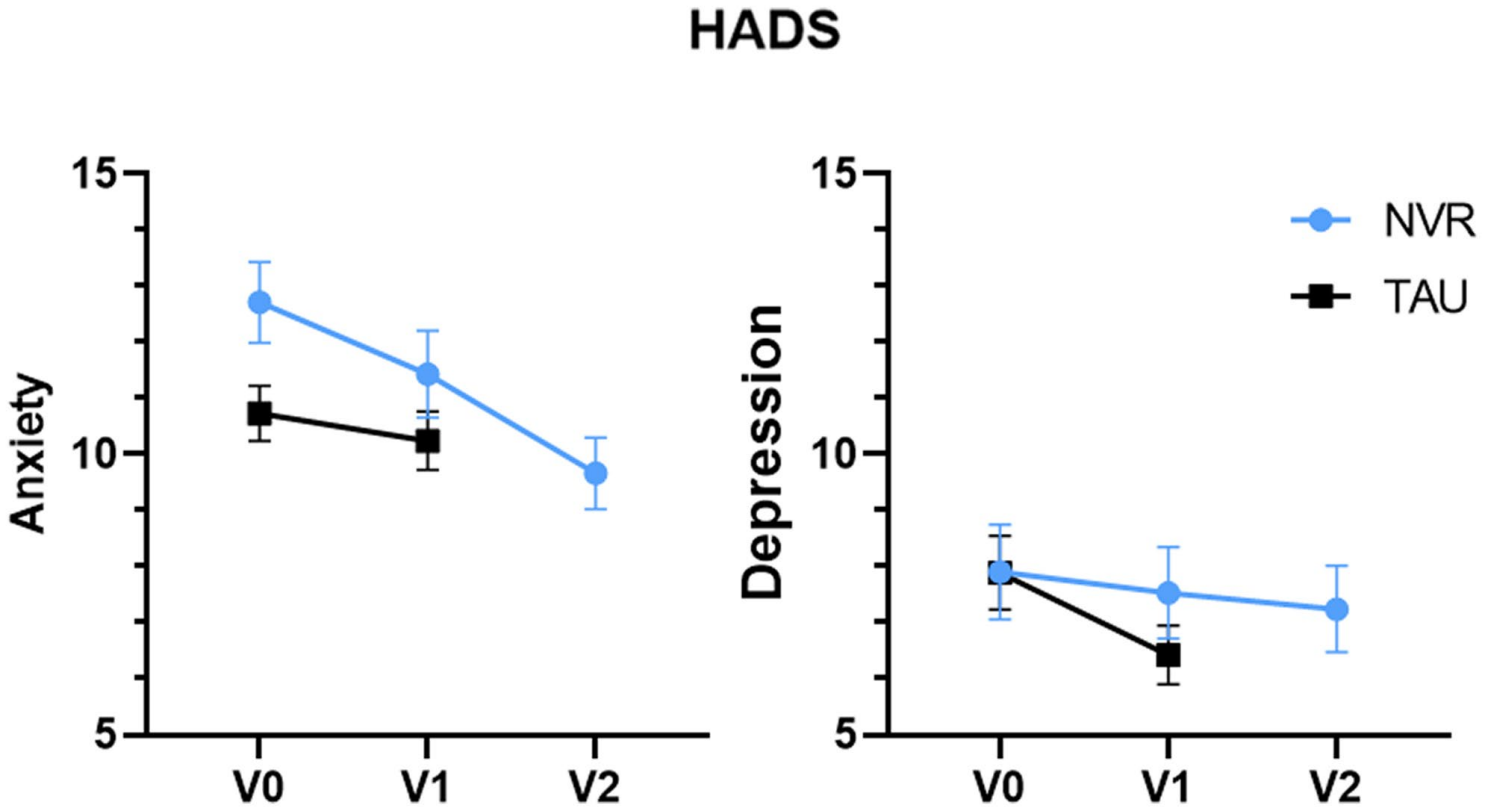
HADS scores. Values of HADS for number of subjects at baseline, V0 (NVR = 31, TAU = 37), at completion, V1 (NVR = 30, TAU = 33) and at post-treatment, V2 (NVR = 29). Values are mean ± standard deviation.

The socio-demographic and clinical results of parents and children are presented in Table 3. There were no socio-demographic differences between the two groups, except for the family financial impact: parents from the NVR group were spending more money on their child, negatively impacting family finances [NVR: 21 (58.3%); TAU: 13 (35.1%)]. Parents of children with STB in the NVR had slightly higher PSI-SF scores than TAU group, with overall high levels of stress in this population (Figure 4). Fathers (mean ± SD: 123.1 ± 18.6) and mothers (120.1 ± 20.0) scores showed similar scores. In the HADS-A, both mothers (11.6 ± 3.8) and fathers (9.0 ± 3.9) showed, respectively, definite and doubtful symptomatology of anxiety. In fact, the largest percentage of mothers had definite symptomatology in each group (NVR: 68.7%; TAU: 45.9%). Although a small number of fathers participating in the study, most of them were in the definite symptomatology in NVR (60.0%) and no symptomatology in TAU (66.7%). HADS-A score in NVR was higher at baseline compared to the control group (Figure 5). Overall, In HADS-D, both mothers (7.9 ± 4.4) and fathers (7.4 ± 4.1) showed no symptomatology of depression (Figure 5). Specifically, the largest percentage of mother had no or doubtful symptomatology in each group (NVR: 60.0%; TAU: 51.3%). Most of fathers were in the no symptomatology in NVR (60.0%) and doubtful symptomatology in TAU (66.7%). There were no significant between-group differences at baseline in the scores of parental questionnaires (Table 4). The teacher-reported SDQ differed significantly at baseline: TAU had higher scores in the total difficulties and hyperactivity/inattention dimensions (Supplementary Table S1).

**Table 3:** Demographic and clinical baseline characteristics of NVR and TAU groups.

	Total (*N* = 73)	NVR (*N* = 36)	TAU (*N* = 37)
Measure	n (%)	n (%)	n (%)
Child sex (male)	42 (57.32)	22 (61.11)	20 (54.05)
Child age (mean ± SD)	11.71 (± 2.89)	12.11 (± 2.70)	11.32 (± 3.06)
Child psychiatric/psychological care	61 (84.72)	30 (85.71)	31 (83.78)
Participation			
Parent (mother)	52 (71.23)	27 (75.00)	25 (67.57)
Both parents	19 (26.03)	11 (29.73)	8 (22.22)
Tyrannical behavior (child)			
Afraid of your child	21 (28.77)	26 (72.22)	26 (70.27)
Children decisional power	61 (83.56)	30 (83.33)	31 (83.78)
Violence toward you	70 (95.89)	35 (97.22)	35 (94.59)
Feeling ashamed by this relationship	47 (64.38)	21 (58.33)	26 (70.27)
Financial impacts	39 (53.42)	21 (58.33)	13 (35.14)

**Table 4:** CBCL and SDQ parent-report scores.

CBCL	NVR (*N* = 36)	TAU (*N* = 37)	Abs. diff.* [95%CI]	*P*
Withdrawn/Depressed				
V0	69.47 (± 11.11)	67.19 (± 9.43)	2.28 [−2.52;7.09]	0.35
V1	66.79 (± 9.21)	64.41 (± 11.00)	2.39 [−2.44;7.21]	0.33
V2	66.68 (± 10.67)			
Somatic complaints				
V0	65.11 (± 10.88)	67.49 (± 11.67)	−2.38 [−7.64;2.89]	0.21
V1	63.15 (± 10.51)	66.35 (± 14.49)	−3.20 [−9.24;2.84]	0.29
V2	62.74 (± 10.12)			
Anxious/Depressed				
V0	70.64 (± 10.05)	72.54 (± 11.31)	−1.90 [−6.90;3.10]	0.45
V1	68.47 (± 10.53)	69.86 (± 11.66)	−1.39 [−6.67;3.88]	0.60
V2	65.00 (± 9.09)			
Social problems				
V0	66.00 (± 14.69)	67.05 (± 12.59)	−1.05 [−7.43;5.33]	0.74
V1	64.85 (± 15.57)	61.43 (± 14.02)	3.42 [−3.59;10.43]	0.33
V2	64.81 (± 11.31)			
Thought problems				
V0	65.72 (± 10.83)	66.70 (± 8.77)	−0.98 [−5.57;3.61]	0.67
V1	64.62 (± 9.91)	62.35 (± 17.02)	2.27 [−4.28;8.82]	0.50
V2	63.61 (± 9.87)			
Attention problems				
V0	71.58 (± 11.55)	69.70 (± 13.99)	1.88 [−4.11;7.88]	0.53
V1	69.53 (± 10.57)	67.84 (± 15.12)	1.69 [−4.45;7.84]	0.59
V2	66.71 (± 10.92)			
Conduct problems				
V0	70.14 (± 6.56)	68.32 (± 12.30)	1.81 [−2.79;6.42]	0.44
V1	63.41 (± 17.09)	64.03 (± 16.23)	−0.62 [−8.51;7.28]	0.88
V2	66.35 (± 9.28)			
Aggressive behavior				
V0	76.17 (± 10.38)	73.30 (± 14.60)	2.87 [−3.04;8.78]	0.34
V1	72.56 (± 12.30)	68.03 (± 14.54)	4.53 [−1.88;10.94]	0.16
V2	71.19 (± 12.16)			
Dysregulation profile				
V0	218.39 (± 25.34)	215.54 (± 30.42)	2.85 [−10.24;15.93]	0.67
V1	210.56 (± 25.70)	205.73 (± 33.88)	4.83 [−9.50;19.16]	0.50
V2	202.90 (± 27.68)			
Total problems				
V0	74.64 (± 6.52)	75.51 (± 6.91)	−0.87 [−4.01;2.26]	0.58
V1	70.41 (± 14.04)	69.49 (± 14.48)	0.93 [−5.84;7.69]	0.79
V2	69.55 (± 9.93)			
Internalizing problems				
V0	71.72 (± 8.45)	72.76 (± 8.67)	−1.03 [−5.03;2.96]	0.61
V1	69.26 (± 8.70)	70.30 (± 10.97)	−1.03 [−5.75;3.68]	0.66
V2	66.97 (± 9.41)			
Externalizing problems				
V0	74.17 (± 7.99)	73.35 (± 5.23)	0.82 [−2.36;3.99]	0.61
V1	70.88 (± 8.57)	68.89 (± 8.71)	1.99 [−2.11;6.09]	0.34
V2	69.23 (± 10.27)			
SDQ parent report	NVR	TAU	Abs. diff.* [95%CI]	*P*
Emotional symptoms				
V0	4.86 (± 2.80)	6.03 (± 2.57)	−1.17 [−2.42;0.09]	0.07
V1	4.70 (± 2.26)	5.44 (± 2.80)	−0.75 [−1.98;0.48]	0.23
V2	4.45 (± 2.75)			
Conduct problems				
V0	5.33 (± 1.87)	5.46 (± 1.97)	−0.13 [−1.02;0.77]	0.78
V1	5.58 (± 2.39)	4.56 (± 2.03)	1.02 [−0.04;2.08]	0.06
V2	4.71 (± 2.27)			
Hyperactivity-inattention				
V0	6.08 (± 3.25)	6.43 (± 2.84)	−0.35 [−1.77;1.07]	0.63
V1	6.27 (± 2.27)	6.03 (± 2.74)	0.24 [−0.97;1.46]	0.69
V2	5.71 (± 2.62)			
Peer relationship problems				
V0	4.31 (± 2.39)	3.86 (± 2.39)	0.44 [−0.68;1.56]	0.43
V1	3.85 (± 2.51)	3.50 (± 2.25)	0.35 [−0.80;1.49]	0.54
V2	4.19 (± 2.54)			
Total difficulties				
V0	20.58 (± 6.61)	21.78 (± 6.59)	−1.20 [−4.28;1.88]	0.44
V1	20.39 (± 5.51)	19.53 (± 6.84)	0.87 [−2.14;3.87]	0.57
V2	19.06 (± 6.96)			
Prosocial behaviors				
V0	4.97 (± 3.03)	5.51 (± 2.79)	−0.54 [−1.91;0.83]	0.43
V1	5.33 (± 2.88)	5.74 (± 2.69)	−0.41 [−1.76;0.94]	0.55
V2	5.97 (± 2.94)			
Child’s distress				
V0	1.39 (± 0.84)	1.35 (± 0.72)	0.04 [−0.33;0.40]	0.84
V1	1.18 (± 0.77)	1.31 (± 0.80)	−0.13 [−0.51;0.25]	0.49
V2	1.26 (± 0.89)			
Learning impact				
V0	1.36 (± 0.80)	1.03 (± 0.90)	0.33 [−0.06;0.73]	0.10
V1	1.36 (± 0.86)	0.97 (± 0.89)	0.39 [−0.03;0.82]	0.07
V2	1.48 (± 0.77)			
Family impact				
V0	1.81 (± 0.47)	1.81 (± 0.52)	−0.01 [−0.24;0.23]	0.54
V1	1.85 (± 0.44)	1.59 (± 0.70)	0.26 [−0.03;0.55]	0.07
V2	1.74 (± 0.58)			
Friendship impact				
V0	1.17 (± 0.88)	1.14 (± 0.86)	0.03 [−0.37;0.44]	0.88
V1	1.18 (± 0.88)	0.74 (± 0.85)	0.44 [0.02;0.86]	0.04
V2	1.26 (± 0.86)			
Leisure activities impact				
V0	1.17 (± 0.91)	0.86 (± 0.82)	0.30 [−0.10;0.71]	0.14
V1	1.12 (± 0.86)	0.76 (± 0.83)	0.36 [−0.05;0.78]	0.08
V2	1.23 (± 0.84)			
Impact score				
V0	6.89 (± 2.90)	6.19 (± 2.58)	0.70 [−0.58;1.98]	0.28
V1	6.70 (± 2.21)	5.14 (± 3.10)	1.56 [0.25;2.86]	0.02
V2	6.97 (± 2.63)			

Values of CBCL for number of subjects at baseline, V0 (NVR = 36, TAU = 37), at completion, V1 (NVR = 34, TAU = 37) and at post-treatment, V2 (NVR = 31). Values of SDQ for number of subjects at baseline, V0 (NVR = 36, TAU = 37), at completion, V1 (NVR = 33, TAU = 35) and at post-treatment, V2 (NVR = 31). Values are mean (± standard deviation). *Hodges-Lehmann Method was used to calculate the absolute difference of the mean with its confidence interval.

### Primary outcome

Between-group comparison of the change in the total score of PSI-SF at completion was not significant: NVR mean (SD) −4.3 (13.9) vs. TAU mean (SD) −7.6 (19.6) two-sample *t*-test *p* = 0.42 (Figure 4) with an effect size [95% CI] of −0.19 [−0.67, 0.28]. In the mixed model, the value of p of time effect in NVR group was *p* < 0.001.

### Secondary outcomes

#### Parental functioning

Within-group differences between baseline and completion of PSI-SF total score were significant for TAU (TAU: *p* = 0.03; NVR: *p* = 0.08). However, the NVR PSI-SF scores decreased significantly between baseline and 8-month follow-up (*p* = 0.002) and between completion (4-month) and 8-month follow-up (*p* = 0.03) (Figure 4).

Between-group comparison of the change of HADS-A (NVR: −1.32 ± 3.11; TAU −0.60 ± 3.85; two-sample *t*-test *p* = 0.41 with an effect size of 0.20 [−0.28, 0.69]) and HADS-D (NVR: −0.13 ± 3.92; TAU: −1.43 ± 3.26; two-sample *t*-test *p* = 0.15) were not significant with an effect size of −0.36 [−0.85, 0.13]. Within-group, HADS-A significantly decreased during time in NVR (baseline vs. completion: *p* = 0.02; completion vs. follow-up: *p* = 0.04; baseline vs. follow-up: *p* < 0.001). HADS-D score showed minor depressive symptoms at baseline for both groups and it decreased progressively, showing significant improvements only in TAU (*p* = 0.02) (Figure 5).

#### Child’s functioning

**Figure 6:**
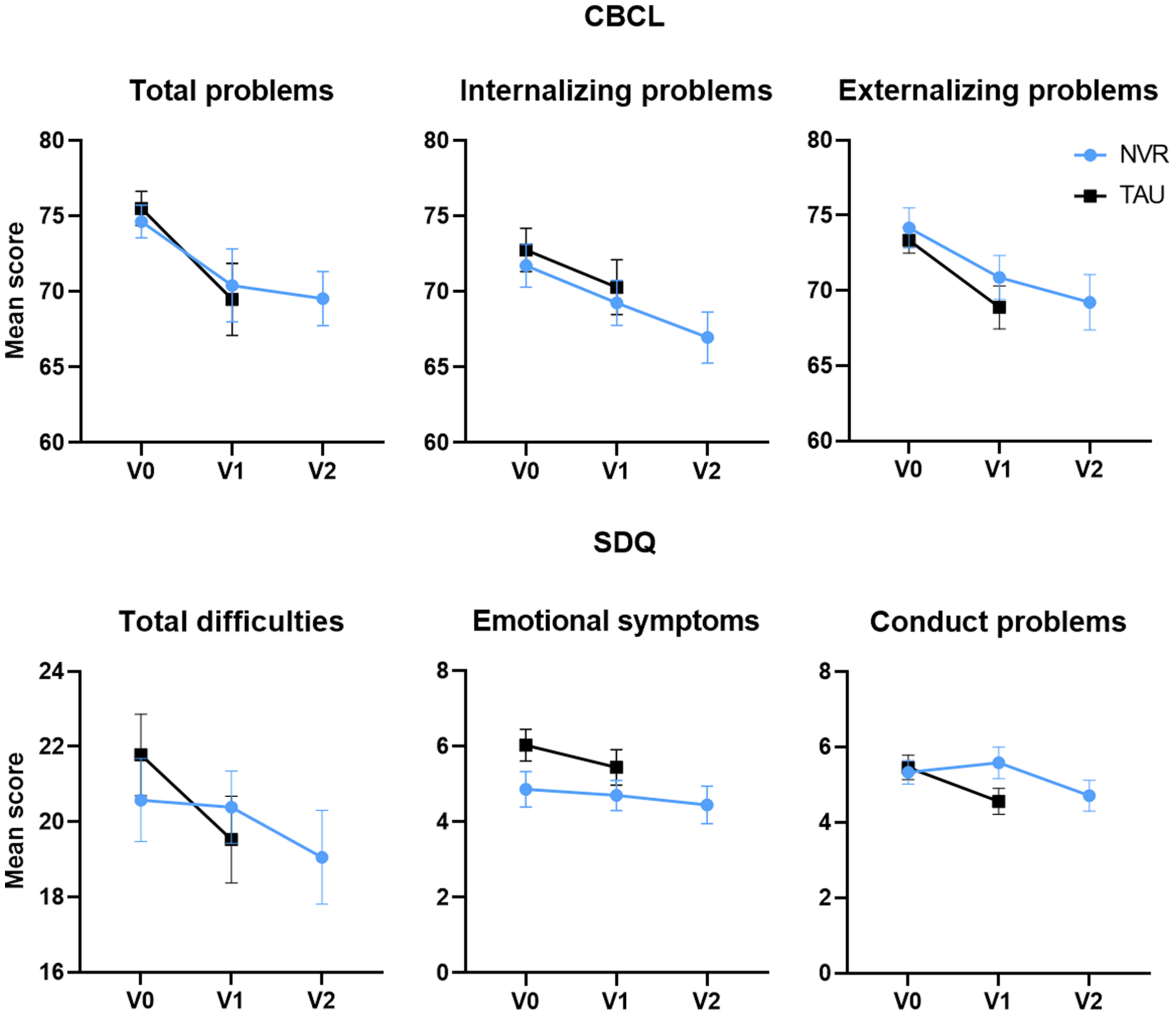
CBCL and parent-reported SDQ scores at baseline and completion. Values are mean ± standard deviation.

Children’s functioning was assessed by parental questionnaires with the CBCL and the SDQ. At baseline, on average the child’s behavior, assessed by CBCL, showed impairments in all domains, with a borderline range (over 95th percentile, T scores 65–70) for depressed-withdrawn, somatic complaints, social problems, thought problems, conduct problems and scores in the clinical range (over 98th percentile) for anxious- depressive, attention problems, aggressive behavior, conduct problems (Table 4) and internalizing and externalizing problems. Symptom scores tended to decrease at completion in both groups (Figure 6). Scores (Table 4) continued this tendency also at follow-up for NVR. Between-group comparison of the change in the CBCL total score and the internalizing and externalizing problem scales showed no significant differences (total score: *p* = 0.68, effect size −0.10 [−0.56, 0.37]; internalizing problem: *p* = 0.67, effect size 0.10 [−0.36, 0.57]; externalizing problem: *p* = 0.61, effect size −0.12 [−0.59, 0.34]) (Table 5). The parent-reported SDQ, used in this study as an additional assessment of behavioral problems, showed borderline and abnormal scores in all domains (Table 4). Between-group comparison of the change in the clinical subscales of SDQ were not significant (total difficulties: *p* = 0.18, effect size −0.33 [−0.80, 0.15]; behavioral problems: *p* = 0.05, effect size −0.48 [−0.95, 0.01]; emotional problems: *p* = 0.23, effect size −0.29 [−0.76, 0.19]) (Table 5). The small number of questionnaires completed by the teachers did not allow comparability at completion (baseline: NVR = 11, TAU = 14; completion: NVR = 4, TAU = 5). Regarding the NVR group, in the mixed model, for the CBCL, the value of p of group effect is *p* = 0.97, time effect was *p* = 0.01 and the time effect for the SDQ was not significant (*p* = 0.11).

**Table 5:** Between-group comparison of the change of the CBCL and SDQ parent-report scores.

CBCL	NVR (*N* = 34)	TAU (*N* = 37)	*p*
Total problems			
V1-V0	−4.76 (± 11.32)	−6.03 (± 14.57)	0.69
Internalizing problems			
V1-V0	−3.21 (± 6.26)	−2.46 (± 8.08)	0.67
Externalizing problems			
V1-V0	−3.47 (± 8.85)	−4.46 (± 7.29)	0.61
SDQ parent report	NVR (*N* = 33)	TAU (*N* = 36)	*P*
Emotional symptoms			
V1-V0	−0.18 (± 1.72)	−0.72 (± 1.99)	0.23
Conduct problems			
V1-V0	−0.24 (± 1.98)	−0.86 (± 2.59)	0.05
Total problems			
V1-V0	−0.55 (± 5.51)	−2.47 (± 6.21)	0.18

Values of CBCL for number of subjects at baseline, V0 (NVR = 36, TAU = 37), at completion, V1 (NVR = 34, TAU = 37) and at post-treatment, V2 (NVR = 31). Values of SDQ for number of subjects at baseline, V0 (NVR = 36, TAU = 37), at completion, V1 (NVR = 33, TAU = 35) and at post-treatment, V2 (NVR = 31). Values are mean (± standard deviation).

## Discussion

This randomized controlled trial evaluated for the first time the efficacy of NVR in reducing parental stress in a specific type of CPV population, described as STB, using TAU as a comparison group. Additionally, as secondary outcomes, the study investigated the efficacy of NVR on parental anxiety and depression, children’s behavioral symptoms and change in clinical variables at follow-up.

### Interpretation

#### Parent related changes

The baseline characteristics of parents of children with STB showed high stress and anxiety levels. Stress and anxiety are family characteristics that have already been associated with CPV (2, 49, 50). We considered the measure of parental stress an indicator of NVR’s efficacy, because this program aims to support parents in modifying the family dynamic, and gradually regain control over the child. Contrarily to our hypothesis, NVR was not superior to TAU for reducing parental stress immediately following the program. This is in line with the data of Weinblatt and Omer (27) where NVR therapy was not effective on overall levels of parental distress (as measured by the Mental health inventory) (51). Unfortunately, we could not compare NVR and TAU at follow-up due to ethical and organizational reasons. Although NVR was not more effective than TAU, within each group, a significant stress reduction in parental stress was shown in TAU at completion and in NVR at follow-up. TAU is a strong comparison condition, capable to reduce the effect of active conditions, and often includes some potentially effective elements that may be absent in no-treatment controls (e.g., positive encouragement) (52, 53). The decrease of parental stress in TAU group may be due to a positive expectancy effect in TAU group, confirmed also by the complete fade of depression in this group. Indeed, these parents knew they would participate in NVR after the end of the program, outside the study protocol: the hope of improvement may explain the parental stress reduction. Parents of the NVR group faced the challenge to change their attitudes and were asked to implement new strategies which could lead to family distress at first and then increase parental stress reliefs. The delay in changes in parental stress seen in NVR seems to indicate that actively implementing new family dynamics requires effort and does not lead to rapid changes in the child’s behavior. Changes in parental stress occur at a later stage, possibly related to the long-term effects of program-related modifications in the family interactions or thanks to non-specific effects such as the support of the parent group. Further insights into parents’ experiences, facilitators and obstacles to NVR strategies implementation could certainly be gained from qualitative interviews planned to do in an upcoming study. Moreover, in view of the severity of behavioral problems in this population, individual sessions could be more effectives that group sessions.

An adaptation of the NVR program was previously found effective as a parent training for childhood anxiety disorders (26), improving child anxiety and reducing family accommodation. Although our intervention was focused on children with STB having a range of different emotional and behavioral disorders, the most rapid benefit on anxiety symptoms in this study was directly on the parent. Furthermore, parents of children with STB appeared to have low depression symptoms in contrast with findings in parents of children with an oppositional defiant disorder or conduct disorder (54, 55). Anxiety levels were similar to those found in oppositional defiant disorder (55).

Participation rate was high and dropouts were extremely low, showing the same high treatment acceptance rate and satisfaction as other NVR programs (27). The prevalence of mother participation is in line with participants’ characteristics in parent training programs (56–59).

#### Child related changes

At completion, NVR was not superior at TAU for children’s behavioral and emotional problems as measured by the parent-rated CBCL and SDQ. We found no evidence that the groups changed differently in the CBCL total score and in externalizing problems. When considering within-group changes, NVR showed improvements also in internalizing problems, such as mood disturbance, anxiety, depression, and social withdrawal, while TAU showed improvements in the SDQ total difficulties and in the emotional problems scores. We consider that helping children with STB is a long process and that it will be difficult to get a significant change in a child’s functioning in such short timeframes. Therefore, the children’s improvement throughout parental therapies should be addressed at longer follow-ups.

### Limitation

The current study must be considered in light of several limitations. First, NVR was compared to TAU in reducing parental stress, and not to other active and protocolized intervention programs. This leaves the possibility of expectation effects on parental stress reduction in TAU. Moreover, due to ethical reasons, no follow-up assessments were conducted with TAU to confirm that the changes in NVR at follow-up were due to intervention alone. We also consider the short timeframes we used to be a limitation, when the implementation of parental strategies may need some time to unfold and show effects on children’s behavior.

Furthermore, as suggested by the extremely low dropout rate, there might have been a selection bias in our sample, as parents participating in our study were in active search of help, unlike hard-to-reach probably more reluctant to engage in research programs.

### Generalizability

In this RCT, contrary to our expectation, we have shown that NVR was not superior to TAU in reducing parental stress at completion of the NVR program for parents of children with STB. However, NVR showed positive outcomes in the follow-up period, pointing to the importance to investigate possible barriers in implementing parental strategies and following this population over longer time periods in future projects. Follow-up assessments are necessary to investigate the possible long-term effects of NVR in the prevention of negative developmental outcomes, although it could be difficult to conduct such studies without adequate control conditions (60). Further studies need to investigate different interventions and treatment outcomes in children with STB, as well as mediators and moderators of treatment effects to understand the mechanisms as this population is understudied despite parental distress and anxiety, and complex symptoms in children. Nowadays, the principal treatment for parent abuse remains family therapy (61) and these data highlight the importance of implementing health care supports and parent therapies like NVR not only for parents but also for child psychological support. At this juncture, it seems essential to better characterize and evaluate the population of children with STB in future studies.

## Data availability statement

The raw data supporting the conclusions of this article will be made available by the authors, without undue reservation.

## Ethics statement

The studies involving human participants were reviewed and approved by Comite de Protection des Personnes Ouest III. Written informed consent to participate in this study was provided by the participants’ legal guardian/next of kin.

Written informed consent was obtained from the individual(s), and minor(s)’ legal guardian/next of kin, for the publication of any potentially identifiable images or data included in this article.

## Author contributions

EF drafted the manuscript. EF, M-CP, NF, SA, and DP-O contributed to the data analysis and interpretation of the data under supervision of NF. NF and DP-O contributed to the design and together with FP to the realization of the program. All authors reviewed and approved the submitted version of the manuscript.

## Funding

This project was funded by a regional grant AOI Montpellier-Nimes 2016. The promoter was the CHU of Montpellier.

## Conflict of interest

During the last 3 years, DP-O has received speaker/consultant honoraria and travel support from Medice and non-financial support from HAC Pharma, unrelated to the current study.

The remaining authors declare that the research was conducted in the absence of any commercial or financial relationships that could be construed as a potential conflict of interest.

## Publisher’s note

All claims expressed in this article are solely those of the authors and do not necessarily represent those of their affiliated organizations, or those of the publisher, the editors and the reviewers. Any product that may be evaluated in this article, or claim that may be made by its manufacturer, is not guaranteed or endorsed by the publisher.

## Abbreviations

ADHD: attention deficit hyperactivity disorder
CPV: child-to-parent violence
STB: severe tyrannical behavior
