# Supplementary material for: Non-violent resistance parental training versus treatment as usual for children and adolescents with severe tyrannical behavior: a randomized controlled trial
Source: Front Psychiatry. 2023 May 4;14:1124028. doi: 10.3389/fpsyt.2023.1124028 (PMC10195028; doi:10.3389/fpsyt.2023.1124028)
Supplement: Supplementary file 1 [file Data_Sheet_1.pdf]

SUPPLEMENTARY MATERIALS

TITLE

Non-Violent Resistance parental training versus treatment as usual for children and adolescents with severe tyrannical behaviour: a randomized controlled trial

Erica Fongaro[1,2], Safa Aouinti[3], Marie-Christine Picot[3], Florence Pupier[1], Haim Omer[4], Nathalie Franc[1] / Diane Purper-Ouakil[1,2]

[1] Centre Hospitalier Universitaire de Montpellier, Saint Eloi Hospital, Montpellier, France

[2] CESP INSERM U 1018 UVSQ Psychiatry Development and Trajectories, Villejuif, France

[3] Centre Hospitalier Universitaire de Montpellier, Unité de Recherche Clinique & Epidémiologie, DIM, Montpellier, Hérault, France

[4] Department of Psychology, Tel Aviv University, 69 978, Ramat-Aviv, Israel

*corresponding author: Erica Fongaro

Email address: erica.fongaro@chu-montpellier.fr

Journal
Frontiers in Psychiatry

Table 1: Teacher-reported SDQ. Values of teacher-reported SDQ for number of subjects at baseline, V0 (NVR=11, TAU=5), at completion, V1 (NVR=11, TAU=5) and at post-treatment, V2 (NVR=11). Values are mean (± standard deviation).

**SDQ Teacher Report**	**NVR**	**TAU**	**p**
Emotional symptoms			
V0	2.64 (±1.29)	4.29 (±3.52)	0.15
V1	1.00 (± 1.00)	4.20 (± 3.77)	0.13
V2	4.22 (± 2.64)		
Conduct problems			
V0	1.27 (±0.90)	2.14 (±2.07)	0.17
V1	0.80 (± 0.84)	2.40 (± 1.95)	0.13
V2	2.78 (± 2.28)		
Hyperactivity-inattention			
V0	3.73 (±2.28)	6.21 (±2.78)	0.03
V1	4.00 (± 2.83)	7.20 (± 0.45)	0.04
V2	4.44 (± 2.07)		
Peer relationship problems			
V0	1.73 (±1.68)	3.21 (±2.64)	0.12
V1	1.75 (± 1.50)	3.00 (± 2.55)	0.42

V2	3.44 (± 1.67)		
Total Difficulties			
V0	9.36 (±4.15)	15.86 (±8.64)	0.02
V1	7.00 (± 5.72)	16.80 (± 7.36)	0.07
V2	14.89 (± 5.16)		
Prosocial behavior			
V0	7.50 (±2.42)	5.21 (±3.19)	0.07
V1	6.75 (± 3.30)	5.00 (± 1.58)	0.33
V2	5.86 (± 3.13)		
Child’s distress			
V0	0.67 (±0.87)	1.14 (±0.86)	0.21
V1	0.67 (± 0.58)	1.20 (± 1.10)	0.47
V2	0.89 (± 0.78)		
Learning impact			
V0	0.44 (±0.73)	0.93 (±1.00)	0.22
V1	0.67 (± 1.15)	1.60 (± 0.55)	0.16
V2	0.67 (± 0.87)		
Friendship impact			
V0	0.38 (±0.74)	0.46 (±0.66)	0.78
V1	0.33 (± 0.58)	1.25 (± 0.96)	0.21
V2	0.89 (± 0.93)		
Impact score			
V0	1.18 (±1.78)	2.50 (±2.03)	0.10
V1	1.00 (± 1.73)	3.80 (± 1.92)	0.04
V2	2.44 (± 2.35)		
